# Nitrogen and boron nutrition in grafted watermelon II: Impact on nutrient accumulation in fruit rind and flesh

**DOI:** 10.1371/journal.pone.0252437

**Published:** 2021-05-27

**Authors:** Kemal Yalçın Gülüt, Ebru Duymuş, İlknur Solmaz, Ayfer Alkan Torun

**Affiliations:** 1 Faculty of Agriculture, Department of Soil Science and Plant Nutrition, Çukurova University, Adana, Turkey; 2 Faculty of Agriculture, Department of Horticulture, Çukurova University, Adana, Turkey; Harran Üniversitesi: Harran Universitesi, TURKEY

## Abstract

Turkey ranks second in watermelon (*Citrullus lunatus* L.) production globally and the highest production is witnessed for Çukurova plains the country. Although watermelon is extensively cultivated in the Çukurova region, studies on optimum nitrogen (N) and boron (B) doses for watermelon cultivation are quite limited. This study, evaluated the impact of increasing N (0, 90, 180 and 270 kg ha^-1^) and B (0 and 2 kg ha^-1^ B) doses on nutrient uptake in rind (exocarp) and flesh (endocarp) of watermelon fruit. Grafted watermelon variety ‘Starburst’, widely cultivated in the region was used as experimental material. The concentrations of different macro and micronutrients were analyzed from fruit rind and flesh. Individual and interactive effect of N and B doses significantly altered macro and micronutrients’ uptake in rind and flesh. Higher amounts of macro and micronutrients were accumulated in rind than flesh. Nutrients’ uptake was increased with increasing N doses, whereas B had limited impact. The accumulated nutrients were within the safe limits for human consumption. The N concentrations of rind and flesh increased with increasing N dose. Similarly, B concentration in rind and flesh and N concentration in rind significantly increased, while N concentration in flesh decreased with B application. It was concluded that 270 kg ha^-1^ N and 2 kg ha^-1^ B are optimum for better nutrient uptake in watermelon fruit. Thus, these doses must be used for watermelon cultivation in Çukurova plains of the country.

## Introduction

Watermelon (*Citrullus lanatus* L.) is globally important fruit vegetable cultivated on commercial level. China is the leading watermelon producer in the world followed by Turkey [1]. Watermelon is extensively cultivated in Turkey and the country follows China with 10% share in global watermelon production. According to Turkish Ministry of Food, Agriculture and Livestock, annual watermelon production in Turkey is 3.9 million tons. Watermelon production is adversely affected by numerous factors and mineral nutrition is among the major reasons of low yield [1,2]. Nitrogen (N) and boron (B) are critical nutrients required for optimum watermelon production [1–5].

Nitrogen is a macronutrient and required in large amount for normal growth and development of crop plants. Numerous metabolic and biochemical process in plants require N for proper development and higher yield. Chlorophyll formation and photosynthates’ assimilation are directly influenced by N [6–10]. Low N availability hampers plant growth as it is an important constituent of amino acids, nucleic acid, proteins, chlorophyll and hormones [11]. Nonetheless, plant architecture, photosynthesis, flowering and fruit development are positively influenced by optimum N availability resulting in higher yields [6,12,13]. Plant roots absorb N either in nitrate (NO_3_^−^) or in ammonium (NH_4_^+^) form. The NO_3_^−^ is transformed to NH_4_^+^ and subsequently NH_4_^+^ is converted to glutamine or glutamate. The compounds that are synthesized in this process are utilized as a precursor in the formation of amino acids, proteins and other N-containing metabolites [6,7]. Nitrogen is the most deficient nutrient in plant production and important for increasing yield. Nonetheless, excess N supply causes late ripening of fruits, leading to a decreased resistance to certain diseases [14,15]. Therefore, determining and supplying optimum N is imperative for successful crop production and higher economic returns.

Boron is required at all developmental stages of crop plants; however, fruit development is the most critical stage [16]. The cultivated soils of the world are very low in B [17]. Boron fertilizers are frequently used to overcome B-deficiency; however, their excessive application could cause B toxicity. Boron deficiency causes vegetative and reproductive defects in plants; therefore, it must be supplied in sufficient quantities. Chlorosis and thick curled leaves with water soaked black spots are typical B-deficiency symptoms in watermelon [5]. The plants capable of accumulating B under B-deficit conditions are well adapted to the soils low in B [18].

Special attention should be given to B nutrition in areas with high relative humidity [19]. Boron deficiency symptoms gradually increase and become fully visible during flowering phase in watermelon [20]. It is well-known that B plays a critical role during reproductive phase compared to vegetative period of plants [21]. Boron must be supplied to plants during flowering and fruit/grain formation in order to harvest higher yields [22].

Nutrient uptake is critical for the proper growth and development of crop plants. Nutrient use efficiency (NUE) can be improved through several approaches. These approaches include modifications in root architecture [23–25], efficient fertilizer application method and soil microorganism [10,26]. Nonetheless, rootstocks are utilized to improve NUE in fruit and vegetable crops [10,26–28]. Rootstock has improved ion uptake in several species [6,27,29–31]. Therefore, selection of an efficient rootstock is important to get high yields [32,33]. Plant biologists are currently working to identify nutrient-specific rootstocks to overcome the deficiency of a particular nutrient [26,32,33]. Watermelon cultivars in Turkey are grafted and well-adapted to Çukurova region. However, limited is known for their optimum N and B requirements.

Watermelon is cultivated in Turkey in open fields and low tunnels. Çukurova region is important watermelon producer [34] and Adana province shares 20% production in the region. Conscious and balanced fertilization is required to obtain higher yield and quality. This study was conducted to optimize N and B doses for nutrient uptake in watermelon. It was hypothesized that nutrient uptake will linearly increase with increasing N and B doses. The optimized doses would help to improve nutrient uptake and productivity of watermelon in the region.

## Materials and methods

### Studied species

Watermelon, a Cucurbitaceae member is a xerophytic tropical fruit. It is widely cultivated in warm regions [35]. Watermelon fruit has a thick rind (exocarp) that has variable pigmentation with a solid or striped appearance, a fleshy mesocarp and an endocarp which varies in color from white to yellow or red [36,37].

### Experimental site

This study was conducted at experimental fields of Research and Application Center, Çukurova University, Faculty of Agriculture, Department of Soil Science and Plant Nutrition, Turkey during 2018 and 2019. Grafted watermelon cultivar ‘Starburst’, widely cultivated in Çukurova region was used in the experiments. The experiments were set up according to split plot design keeping N as main factor and B as sub-factor. All experimental treatments had four replications and edge effect was excluded to possible extent.

Seedlings were planted keeping 4 m distance between rows, 1.2 m between plants and 6 plants were transplanted in each replication. The soil was analyzed prior to experimentation and depending on the results of the soil analysis 25 kg phosphorus (P_2_O_5_) was applied per hectare at the time of planting.

Four different N doses, i.e., N_0_ (0 kg N ha^-1^), N_1_ (90 kg N ha^-1^), N_2_ (180 kg N ha^-1^) and N_3_ (270 kg N ha^-1^) and two different B doses, i.e., B_0_ (0 kg ha^-1^) and B_2_ (2 kg ha^-1^ B) were used in the study. Nitrogen was applied by using ammonium sulfate as a source and applied in three equal splits (i.e., at sowing, flowering and fruiting). Etidot67-B was used as B source and all B was applied at sowing.

The fruits were manually harvested at harvest maturity. For nutrient analysis, samples were washed with distilled water, 0.1% HCl and tap water. After washing, rind and flesh were separated. Separated samples were chopped into small pieces and dried in an oven at 70°C until constant weight. The dried samples were ground in an agate mill, separately for analysis. The ground samples were burnt in ash furnace according to dry burning method [38]. Boron concentration was analyzed on spectrophotometer following Bingham [39]. Nitrogen was analyzed according to Kjeldahl method [40]. The Ca, Mg and K were analyzed by semi-micro wet digestion method [41]. The concentrations of Zn, Fe, Mn and Cu in the digested solutions were determined by inductively coupled plasma atomic emission spectroscopy (ICP-AES, OPTIMA 3300 DV, Perkin-Elmer, USA) [42].

### Statistical analysis

The collected data for nutrient uptake were tested for normality by Shapiro-Wilk normality test [43]. The data were normally distributed; therefore, original data were used in statistical analysis. The differences among years were analyzed by paired t test, which were significant. Therefore, data of both years were analyzed and presented separately. Two-way analysis of variance (ANOVA) was used to test the differences among N and B doses, and their interaction [44]. Least significant difference at 5% probability was used to separate means where ANOVA indicated significant differences. Finally, spearman correlation was computed among nutrient acquisition in rind and flesh, separately. The correlation was computed on PAST software [45].

## Results

The nutrients accumulated by the rind and flesh were divided into macro and micronutrients based on human consumption. Calcium (Ca), magnesium (Mg) and potassium (K) are required in large quantities by humans; therefore, referred as macro elements, whereas iron (Fe), copper (Cu), manganese (Mn), boron (B), zinc (Zn) and N are required in trace/minor amounts; thus, regarded as micro elements.

### Macronutrients’ accumulation in rind

Different N doses significantly (p<0.05) altered macro elements’ concentration in rind during both years, except for K concentration during 1^st^ year (Table 1). Similarly, Mg during 1^st^ year and Ca and K during 2^nd^ year were significantly (p<0.05) affected by B doses, while rest of the macro elements were not affected (p>0.05). Nonetheless, interactive effects of N and B were significant for all of the macro elements in the rind during both years (Table 1).

**Table 1:** Analysis of variance of different mineral uptake traits of grafted watermelon rind grown under various nitrogen and boron doses.

			Year-1				Year-2			
Mineral	SOV	DF	SS	MS	F value	P value	SS	MS	F value	P value
**Ca**	N	3	0.16	0.05	2.21	0.003*	0.13	0.04	7.50	0.00*
	B	1	0.05	0.05	2.09	0.152^NS^	0.04	0.04	7.63	0.01*
	N × B	3	0.06	0.02	0.88	0.453^NS^	0.02	0.01	0.94	0.43^NS^
**Mg**	N	3	0.54	0.18	3.94	0.011^NS^	0.05	0.02	11.12	0.0001*
	B	1	0.23	0.23	5.01	0.028*	0.01	0.01	3.84	0.05*
	N × B	3	0.29	0.10	2.12	0.014*	0.02	0.01	4.42	0.01*
**K**	N	3	2.39	0.80	0.96	0.414^NS^	6.61	2.20	8.06	0.0001*
	B	1	0.36	0.36	0.44	0.510^NS^	1.20	1.20	4.40	0.04*
	N × B	3	2.98	0.99	1.21	0.031^NS^	1.24	0.41	1.51	0.22^NS^
**Fe**	N	3	9338.66	3112.89	2.57	0.059^NS^	774.95	258.32	2.35	0.08^NS^
	B	1	1463.95	1463.95	1.21	0.274^NS^	1467.24	1467.24	13.36	0.00*
	N × B	3	14254.82	4751.61	3.93	0.011*	876.87	292.29	2.66	0.05*
**Mn**	N	3	7035.30	2345.10	2.05	0.113^NS^	57.25	19.08	3.06	0.03*
	B	1	141.06	141.06	0.12	0.726^NS^	14.90	14.90	2.39	0.13^NS^
	N × B	3	7217.67	2405.89	2.10	0.105^NS^	119.65	39.88	6.39	0.00*
**Cu**	N	3	445.06	148.35	4.31	0.007*	36.22	12.07	1.61	0.19^NS^
	B	1	0.21	0.21	0.01	0.937^NS^	67.40	67.40	8.97	0.00*
	N × B	3	395.95	131.98	3.83	0.013*	38.60	12.87	1.71	0.17^NS^
**B**	N	3	652.51	217.50	6.90	0.000*	145.63	48.54	5.06	0.00*
	B	1	134.39	134.39	4.26	0.042*	57.57	57.57	6.01	0.02*
	N × B	3	32.30	10.77	0.34	0.795^NS^	7.15	2.38	0.25	0.86^NS^
**Zn**	N	3	4873.05	1624.35	4.86	0.004	140.71	46.90	3.05	0.03*
	B	1	34.83	34.83	0.10	0.748^NS^	13.19	13.19	0.86	0.36^NS^
	N × B	3	2844.74	948.25	2.83	0.043*	19.88	6.63	0.43	0.73^NS^
**N**	N	3	4.21	1.40	9.23	0.0001*	4.31	1.44	10.78	0.0001*
	B	1	0.42	0.42	2.77	0.09^NS^	0.85	0.85	6.36	0.013*
	N × B	3	0.04	0.01	0.08	0.97^NS^	0.41	0.14	1.03	0.385^NS^

SOV = source of variation, DF = degree of freedom, SS = sum of squares, MS = mean squares

* = significant (p<0.05)

NS = non-significant (p>0.05).

The highest concentrations of all macronutrients in rind were noted for N_3_, whereas the lowest values were recorded for N_0_ during both years (Table 2). Similarly, higher amount of Mg was accumulated under B_2_, during first year compared to no B application. However, higher K accumulation was recorded under no B application during 2^nd^ year, while higher Ca was acquired under B_2_ (Table 2).

**Table 2:** The impact of different nitrogen and boron doses and their interaction on macro mineral contents in grafted watermelon rind.

Treatments	Year-1			Year-2		
	Calcium (%)	Magnesium (%)	Potassium (%)	Calcium (%)	Magnesium (%)	Potassium (%)
**Factor A–Nitrogen (N)**						
**0 kg ha**^**-1**^ **(N**_**0**_**)**	0.38 b	0.55 b	6.29	0.28 bc	0.20 c	3.80 b
**90 kg ha**^**-1**^ **(N**_**1**_**)**	0.42 ab	0.64 ab	6.21	0.26 c	0.23 b	4.39 a
**180 kg ha**^**-1**^ **(N**_**2**_**)**	0.44 ab	0.65 ab	6.28	0.31 b	0.24 b	4.40 a
**270 kg ha**^**-1**^ **(N**_**3**_**)**	0.49 a	0.76 a	5.90	0.36 a	0.27 a	4.43 a
**LSD 0.05**	**0.03**	**0.10**	**NS**	**0.04**	**0.03**	**0.22**
**Factor B–Boron (B)**						
**0 kg ha**^**-1**^ **(B**_**0**_**)**	0.41	0.60 b	6.11	0.28 b	0.23	4.37 a
**2 kg ha**^**-1**^ **(B**_**2**_**)**	0.45	0.70 a	6.23	0.32 a	0.24	4.14 b
**LSD 0.05**	**NS**	**0.09**	**NS**	**0.03**	**NS**	**0.21**
**N × B interaction**						
**N**_**0**_**B**_**0**_	0.38 b	0.57 bc	6.14 ab	0.27 cd	0.19 d	4.01 cd
**N**_**1**_**B**_**0**_	0.41 b	0.61 bc	6.21 ab	0.22 d	0.21 d	4.42 abc
**N**_**2**_**B**_**0**_	0.42 b	0.57 bc	5.97 ab	0.29 bc	0.22 cd	4.66 a
**N**_**3**_**B**_**0**_	0.43 b	0.64 bc	6.09 ab	0.35 ab	0.29 a	4.40 abc
**N**_**0**_**B**_**2**_	0.37 b	0.52 c	6.44 ab	0.29 bc	0.21 d	3.60 d
**N**_**1**_**B**_**2**_	0.43 b	0.66 bc	6.21 ab	0.30 bc	0.26 ab	4.37 abc
**N**_**2**_**B**_**2**_	0.46 ab	0.72 ab	6.56 a	0.34 ab	0.25 bc	4.16 bc
**N**_**3**_**B**_**2**_	0.56 a	0.87 a	5.72 b	0.37 a	0.25 bc	4.45 ab
**LSD 0.05**	**0.10**	**0.15**	**0.35**	**0.03**	**0.04**	**0.29**

Means followed by similar letters within a column are statistically non-significant (p>0.05). NS = non-significant.

Regarding N by B interaction, the highest Ca and Mg were accumulated in rind with N_3_ and B_2_ interaction, while plants grown under N_2_ and B_2_ combination acquired the highest amount of K during 1^st^ year. The lowest macro elements’ accumulation in rind was observed for N_0_ and B_0_ combination (Table 2). During 2^nd^ year, N_3_ and B_2_ combination recorded the highest concentration of Ca, whereas N_3_B_1_ combination acquired the highest amount of Mg. Similarly, the highest K uptake was recorded for N_2_B_0_ combination. The lowest concentration of these nutrients was recorded for N_0_B_0_ interaction during 2^nd^ year of the study (Table 2).

### Macronutrients’ accumulation in flesh

The Mg concentration was significantly (p<0.05) affected by N doses during 1^st^ year, whereas Ca and K were not affected (Table 3). Nitrogen doses had significant effect on Mg and K accumulation during 2^nd^ year, while had non-significant on Ca. Different B does had non-significant impact on K uptake during 1^st^ year and Ca accumulation during 2^nd^ year, whereas remaining macro elements were significantly altered by B doses during both years. Nonetheless, interactive effects of N and B were significant for all macro elements except Ca during both years (Table 3).

**Table 3:** Analysis of variance of different mineral uptake traits of grafted watermelon flesh grown under various nitrogen and boron doses.

			Year-I				Year-II			
Mineral	SOV	DF	SS	MS	F value	P value	SS	MS	F value	P value
**Ca**	N	3	0.000	0.000	1.04	0.381^NS^	0.000	0.000	0.817	0.488^NS^
	B	1	0.003	0.003	36.72	0.0001*	0.000	0.000	1.751	0.189 ^NS^
	N × B	3	0.000	0.000	0.85	0.469^NS^	0.001	0.000	6.567	0.000*
**Mg**	N	3	0.71	0.24	21.75	0.0001*	0.01	0.00	7.65	0.00*
	B	1	0.26	0.26	23.38	0.0001*	0.00	0.00	9.70	0.00*
	N × B	3	0.74	0.25	22.59	0.0001*	0.00	0.00	2.45	0.07^NS^
**K**	N	3	0.008	0.003	0.71	0.551^NS^	2.79	0.93	7.77	0.00*
	B	1	0.002	0.002	0.55	0.461^NS^	2.48	2.48	20.72	0.0001*
	N × B	3	0.019	0.006	1.65	0.184^NS^	0.85	0.28	2.35	0.08^NS^
**Fe**	N	3	197.43	65.81	0.99	0.403^NS^	3978.58	1326.19	5.09	0.00*
	B	1	0.72	0.72	0.01	0.917^NS^	1246.96	1246.96	4.78	0.03*
	N × B	3	465.38	155.13	2.33	0.080^NS^	1457.46	485.82	1.86	0.14^NS^
**Mn**	N	3	32.65	10.88	1.04	0.379^NS^	51.38	17.13	4.18	0.01*
	B	1	15.93	15.93	1.52	0.221^NS^	31.44	31.44	7.66	0.01*
	N × B	3	76.09	25.36	2.42	0.071^NS^	14.24	4.75	1.16	0.33^NS^
**Cu**	N	3	1.87	0.62	0.78	0.51^NS^	1.32	0.44	0.21	0.89^NS^
	B	1	10.93	10.93	13.63	0.000*	0.54	0.54	0.25	0.62^NS^
	N × B	3	2.21	0.74	0.92	0.436^NS^	9.86	3.29	1.53	0.21^NS^
**B**	N	3	20.52	6.84	1.18	0.324^NS^	3.37	1.12	0.74	0.53^NS^
	B	1	9.56	9.56	1.64	0.203^NS^	8.25	8.25	5.47	0.02*
	N × B	3	0.93	0.31	0.05	0.984^NS^	3.54	1.18	0.78	0.51^NS^
**Zn**	N	3	15.86	5.29	0.62	0.604^NS^	36.06	12.02	1.58	0.20^NS^
	B	1	28.30	28.30	3.32	0.072^NS^	14.55	14.55	1.91	0.17^NS^
	N × B	3	40.17	13.39	1.57	0.202^NS^	11.75	3.92	0.51	0.67^NS^
**N**	N	3	2.30	0.77	19.01	0.0001*	1.57	0.52	12.79	0.0001*
	B	1	0.02	0.02	0.38	0.539^NS^	0.92	0.92	22.58	0.0001*
	N × B	3	0.41	0.14	3.36	0.022*	0.01	0.00	0.11	0.95^NS^

SOV = source of variation, DF = degree of freedom, SS = sum of squares, MS = mean squares

* = significant (p<0.05)

NS = non-significant (p>0.05).

The highest Mg concentration was noted for N_3_ during 1^st^ year, whereas N doses were non-significant for the rest of macro elements. Similarly, N_1_ and N_2_ recorded the highest concentrations of Mg and K, respectively during 2^nd^ year of the study (Table 4). Similarly, the highest Ca and Mg concentrations during 1^st^ year and Mg and K concentrations during 2^nd^ year were noted for B_2_ (Table 4).

**Table 4:** The impact of different nitrogen and boron doses and their interaction on macro mineral contents in watermelon flesh.

Treatments	Year-1			Year-2		
	Calcium (%)	Magnesium (%)	Potassium (%)	Calcium (%)	Magnesium (%)	Potassium (%)
**Factor A–Nitrogen (N)**						
**0 kg ha**^**-1**^ **(N**_**0**_**)**	0.09	0.14 b	0.82	0.04	0.15 b	1.65 c
**90 kg ha**^**-1**^ **(N**_**1**_**)**	0.09	0.13 b	0.83	0.04	0.18 a	1.93 b
**180 kg ha**^**-1**^ **(N**_**2**_**)**	0.09	0.15 b	0.83	0.04	0.16 b	2.13 a
**270 kg ha**^**-1**^ **(N**_**3**_**)**	0.09	0.34 a	0.81	0.04	0.16 b	1.90 b
**LSD 0.05**	**NS**	**0.11**	**NS**	**NS**	**0.02**	
**Factor B–Boron (B)**						
**0 kg ha**^**-1**^ **(B**_**0**_**)**	0.08 b	0.13 b	0.82	0.04	0.16 b	1.73 b
**2 kg ha**^**-1**^ **(B**_**2**_**)**	0.09 a	0.24 a	0.82	0.04	0.17 a	2.06 a
**LSD 0.05**	**0.01**	**0.09**	**NS**	**NS**	**0.01**	**0.23**
**N × B interaction**						
**N**_**0**_**B**_**0**_	0.08	0.14 b	0.81 ab	0.04	0.15 b	1.59 e
**N**_**1**_**B**_**0**_	0.08	0.13 b	0.83 ab	0.04	0.16 b	1.81 cde
**N**_**2**_**B**_**0**_	0.08	0.13 b	0.83 ab	0.04	0.15 b	1.97 bcd
**N**_**3**_**B**_**0**_	0.08	0.13 b	0.84 a	0.05	0.16 b	1.58 e
**N**_**0**_**B**_**2**_	0.09	0.13 b	0.82 ab	0.05	0.16 b	1.71 de
**N**_**1**_**B**_**2**_	0.10	0.12 b	0.83 ab	0.04	0.20 a	2.06 abc
**N**_**2**_**B**_**2**_	0.09	0.16 b	0.82 ab	0.04	0.17 b	2.28 a
**N**_**3**_**B**_**2**_	0.09	0.54 a	0.78 b	0.04	0.16 b	2.21 ab
**LSD 0.05**	**NS**	**0.25**	**0.04**	**NS**	**0.02**	**0.05**

Means followed by similar letters within a column are statistically non-significant (p>0.05). NS = non-significant.

Regarding N × B interaction, the highest Mg and K concentrations were recorded for N_3_B_2_ and N_3_B_1_, respectively during 1^st^ year. The lowest accumulation of macro elements in rind was observed for N_0_B_0_ (Table 4). During 2^nd^ year, N_1_B_2_ and N_2_B_2_ recorded the highest concentrations of Mg and K, respectively. The lowest concentration of these nutrients was recorded for N_0_B_0_ during 2^nd^ year (Table 4).

### Microelements’ accumulation in rind

The concentration of all microelements in rind was significantly affected by different N doses during both years except non-significant effect for Cu uptake during 2^nd^ year (Table 1). All microelements, except B were not affected by B doses during first year; however, B doses significantly altered all microelements during 2^nd^ year except Mn and Zn. The N × B interaction had significant effect on the concentration of all microelements during both years (Table 1).

The highest concentration of Fe, Mn and Cu was noted with N_1_, whereas N_3_ recorded the highest concentration of B, Zn and N during 1^st^ year (Table 5). The highest concentration of all microelements was observed for N_2_ and N_3_ during 2^nd^ year. The highest concentration of B was recorded under B_2_, whereas B application had no impact on rest of the microelements during 1^st^ year. Regarding interaction N_3_ with both B doses observed the highest concentration of all microelements, while N_0_B_0_ had the lowest values of these traits during both years (Table 5).

**Table 5:** The impact of different nitrogen and boron doses and their interaction on micro mineral contents in watermelon rind.

Year-I						
Treatments	Iron (mg kg^-1^)	Manganese (mg kg^-1^)	Copper (mg kg^-1^)	Boron (mg kg^-1^)	Zinc (mg kg^-1^)	Nitrogen (mg kg^-1^)
**Factor A–Nitrogen (N)**						
**0 kg ha**^**-1**^ **(N**_**0**_**)**	112.61 a	82.07 ab	13.04 ab	32.98 b	66.41 bc	2.32 b
**90 kg ha**^**-1**^ **(N**_**1**_**)**	118.02 a	89.98 a	16.38 a	33.84 b	73.05 ab	2.38 b
**180 kg ha**^**-1**^ **(N**_**2**_**)**	91.36 b	65.99 b	10.25 b	34.21 b	61.04 c	2.48 b
**270 kg ha**^**-1**^ **(N**_**3**_**)**	109.41 ab	78.62 ab	13.02 ab	39.61 a	80.21 a	2.86 a
**LSD 0.05**	**7.76**	**8.12**	**3.40**	**4.44**	**12.21**	**0.34**
**Factor B–Boron (B)**						
**0 kg ha**^**-1**^ **(B**_**0**_**)**	104.23	78.21	13.19	33.98 b	69.76	2.44
**2 kg ha**^**-1**^ **(B**_**2**_**)**	111.73	80.37	13.22	36.34 a	70.78	2.57
**LSD 0.05**	**NS**	**NS**	**NS**	**2.34**	**NS**	**NS**
**N × B interaction**						
**N**_**0**_**B**_**0**_	100.92 bc	88.79 ab	13.02 bc	31.52 c	62.16 c	2.26 c
**N**_**1**_**B**_**0**_	98.81 bc	75.49 b	13.40 bc	33.08 bc	67.34 bc	2.28 c
**N**_**2**_**B**_**0**_	95.49 bc	62.32 b	10.31 c	33.67 bc	60.25 c	2.41 c
**N**_**3**_**B**_**0**_	120.99 ab	84.94 ab	15.78 ab	37.61 ab	88.50 a	2.81 ab
**N**_**0**_**B**_**0**_	124.31 ab	75.36 b	13.06 bc	34.43 bc	70.67 bc	2.38 c
**N**_**1**_**B**_**0**_	137.23 a	104.46 a	19.37 a	34.59 bc	78.76 ab	2.48 c
**N**_**2**_**B**_**0**_	87.57 c	69.36 b	10.19 c	34.71 bc	61.76 c	2.54 bc
**N**_**3**_**B**_**2**_	97.82 bc	72.30 b	10.26 c	41.61 a	71.92 bc	2.90 a
**LSD 0.05**	**17.80**	**26.23**	**8.98**	**4.23**	**8.78**	**0.09**
**Year-II**						
**Factor A–Nitrogen (N)**						
**0 kg ha**^**-1**^ **(N**_**0**_**)**	63.69 b	10.55 b	25.44	31.58 b	26.09 b	1.22 b
**90 kg ha**^**-1**^ **(N**_**1**_**)**	69.00 ab	12.12 a	26.72	33.53 a	29.44 a	1.38 b
**180 kg ha**^**-1**^ **(N**_**2**_**)**	71.63 a	11.31 ab	25.26	34.56 a	27.35 ab	1.66 a
**270 kg ha**^**-1**^ **(N**_**3**_**)**	67.67 ab	12.57 a	25.24	34.65 a	28.07 ab	1.75 a
**LSD 0.05**	**7.80**	**2.01**	**NS**	**2.21**	**2.28**	**0.38**
**Factor B–Boron (B)**						
**0 kg ha**^**-1**^ **(B**_**0**_**)**	71.89 a	12.04	26.53 a	32.77 b	28.12	1.40 b
**2 kg ha**^**-1**^ **(B**_**2**_**)**	64.11 b	11.25	24.83 b	34.35 a	27.37	1.60 a
**LSD 0.05**	**3.34**	**NS**	**1.12**	**1.90**	**NS**	**0.18**
**N × B interaction**						
**N**_**0**_**B**_**0**_	63.14 b	10.35 bc	25.90 b	30.65 d	27.21 ab	1.05 d
**N**_**1**_**B**_**0**_	74.06 a	11.93 bc	28.61 a	32.41 cd	29.56 a	1.25 cd
**N**_**2**_**B**_**0**_	75.19 a	10.91 bc	25.50 b	34.14 abc	27.69 ab	1.57 ab
**N**_**3**_**B**_**0**_	75.46 a	14.89 a	26.00 b	34.01 abc	27.99 ab	1.76 a
**N**_**0**_**B**_**0**_	64.25 b	10.74 bc	24.99 b	32.50 bcd	24.97 b	1.39 bc
**N**_**1**_**B**_**0**_	63.94 b	12.32 b	24.83 b	34.66 abc	29.32 a	1.51 abc
**N**_**2**_**B**_**0**_	68.37 ab	11.68 bc	25.04 b	34.94 ab	27.03 ab	1.74 a
**N**_**3**_**B**_**2**_	59.88 b	10.24 c	24.48 b	35.29 a	28.15 a	1.74 a
**LSD 0.05**	**11.02**	**1.87**	**3.32**	**1.34**	**2.12**	**0.45**

Means followed by similar letters within a column are statistically non-significant (p>0.05). NS = non-significant.

### Microelement accumulation in flesh

The concentration of all microelements except N in flesh was not affected by N doses during 1^st^ year, whereas Fe, Mn and N were significantly altered by N doses during 2^nd^ year (Table 2). All microelements, except Cu were not affected by B doses during first year; however, B doses significantly altered all microelements during 2^nd^ year except Cu and Zn. The N × B interaction had significant effect on the concentration of all microelements during both years except for B during 1^st^ year and Cu during 2^nd^ year (Table 2).

The highest concentration of N was noted with N_2_ and N_3_ during 1^st^ year (Table 6). The highest concentration of all microelements was observed for N_3_ during 2^nd^ year. The highest concentration of Cu was recorded under B_0_, whereas B application had no impact on rest of the microelements during 1^st^ year. Regarding interaction, N_3_ with both B doses observed the highest concentration of all microelements, while N_0_B_0_ had the lowest values of these traits during both years (Table 6).

**Table 6:** The impact of different nitrogen and boron doses and their interaction on micro mineral contents in watermelon flesh.

Year-I						
Treatments	Iron (mg kg^-1^)	Manganese (mg kg^-1^)	Copper (mg kg^-1^)	Boron (mg kg^-1^)	Zinc (mg kg^-1^)	Nitrogen (mg kg^-1^)
**Factor A–Nitrogen (N)**						
**0 kg ha**^**-1**^ **(N**_**0**_**)**	37.13	16.94	4.46	17.27	14.34	1.39 c
**90 kg ha**^**-1**^ **(N**_**1**_**)**	37.20	15.82	4.25	16.42	13.89	1.60 b
**180 kg ha**^**-1**^ **(N**_**2**_**)**	40.49	17.41	4.46	16.52	15.05	1.72 a
**270 kg ha**^**-1**^ **(N**_**3**_**)**	39.43	16.96	4.65	17.49	14.37	1.79 a
**LSD 0.05**	**NS**	**NS**	**NS**	**NS**	**NS**	**0.14**
**Factor B–Boron (B)**						
**0 kg ha**^**-1**^ **(B**_**0**_**)**	38.61	17.18	4.80 a	16.61	14.95	1.61
**2 kg ha**^**-1**^ **(B**_**2**_**)**	38.48	16.38	4.12 b	17.24	13.87	1.64
**LSD 0.05**	**NS**	**NS**	**0.54**	**NS**	**NS**	**NS**
**N × B interaction**						
**N**_**0**_**B**_**0**_	36.80 ab	16.11 b	4.62 abc	17.09	15.19 a	1.47 b
**N**_**1**_**B**_**0**_	34.56 b	15.86 b	4.54 abc	15.96	15.35 a	1.51 b
**N**_**2**_**B**_**0**_	40.28 ab	19.01 a	4.81 ab	16.16	14.99 a	1.72 a
**N**_**3**_**B**_**0**_	42.94 a	17.90 ab	5.22 a	17.20	14.28 ab	1.75 a
**N**_**0**_**B**_**2**_	37.46 ab	17.77 ab	4.30 bc	17.46	13.50 ab	1.30 c
**N**_**1**_**B**_**2**_	39.84 ab	15.78 b	3.97 c	16.88	12.43 b	1.68 a
**N**_**2**_**B**_**2**_	40.68 ab	15.94 b	4.14 bc	16.85	15.10 a	1.73 a
**N**_**3**_**B**_**2**_	35.92 b	16.01 b	4.07 bc	17.77	14.46 ab	1.84 a
**LSD 0.05**	**2.87**	**3.45**	**1.76**	**NS**	**2.34**	**0.75**
**Year-II**						
**Factor A–Nitrogen (N)**						
**0 kg ha**^**-1**^ **(N**_**0**_**)**	107.85 c	17.84 b	7.32	18.32	13.26	1.50 b
**90 kg ha**^**-1**^ **(N**_**1**_**)**	120.10 ab	19.28 a	7.02	17.96	13.60	1.75 a
**180 kg ha**^**-1**^ **(N**_**2**_**)**	113.80 bc	19.63 a	7.24	18.32	14.37	1.76 a
**270 kg ha**^**-1**^ **(N**_**3**_**)**	124.93 a	19.62 a	7.10	18.47	14.82	1.84 a
**LSD 0.05**	**4.56**	**3.45**	**NS**	**NS**	**NS**	**0.30**
**Factor B–Boron (B)**						
**0 kg ha**^**-1**^ **(B**_**0**_**)**	113.07 b	19.66 a	7.25	17.97 b	14.40	1.81 a
**2 kg ha**^**-1**^ **(B**_**2**_**)**	120.26 a	18.52 b	7.10	18.56 a	13.63	1.61 b
**LSD 0.05**	**8.78**	**2.21**	**NS**	**3.45**	**NS**	**0.34**
**N × B interaction**						
**N**_**0**_**B**_**1**_	97.77 b	18.05 cd	7.63	18.06 ab	14.25 ab	1.58 de
**N**_**1**_**B**_**1**_	116.90 a	19.86 ab	7.06	17.93 b	13.79 ab	1.86 ab
**N**_**2**_**B**_**1**_	112.86 a	20.90 a	6.77	17.97 b	14.64 a	1.87 ab
**N**_**3**_**B**_**1**_	124.75 a	19.94 ab	7.48	17.90 b	14.94 a	1.93 a
**N**_**0**_**B**_**2**_	117.93 a	17.64 d	7.01	18.57 ab	12.27 b	1.42 e
**N**_**1**_**B**_**2**_	123.31 a	18.70 bcd	6.98	17.99 b	13.42 ab	1.64 cd
**N**_**2**_**B**_**2**_	114.67 a	18.46 bcd	7.67	18.63 ab	14.12 ab	1.66 cd
**N**_**3**_**B**_**2**_	125.12 a	19.29 abc	6.73	19.04 a	14.69 a	1.74 bc
**LSD 0.05**	**30.90**	**1.03**	**NS**	**2.21**	**0.56**	**0.54**

Means followed by similar letters within a column are statistically non-significant (p>0.05). NS = non-significant.

### Correlation among mineral uptake traits of rind

**Fig 1:**
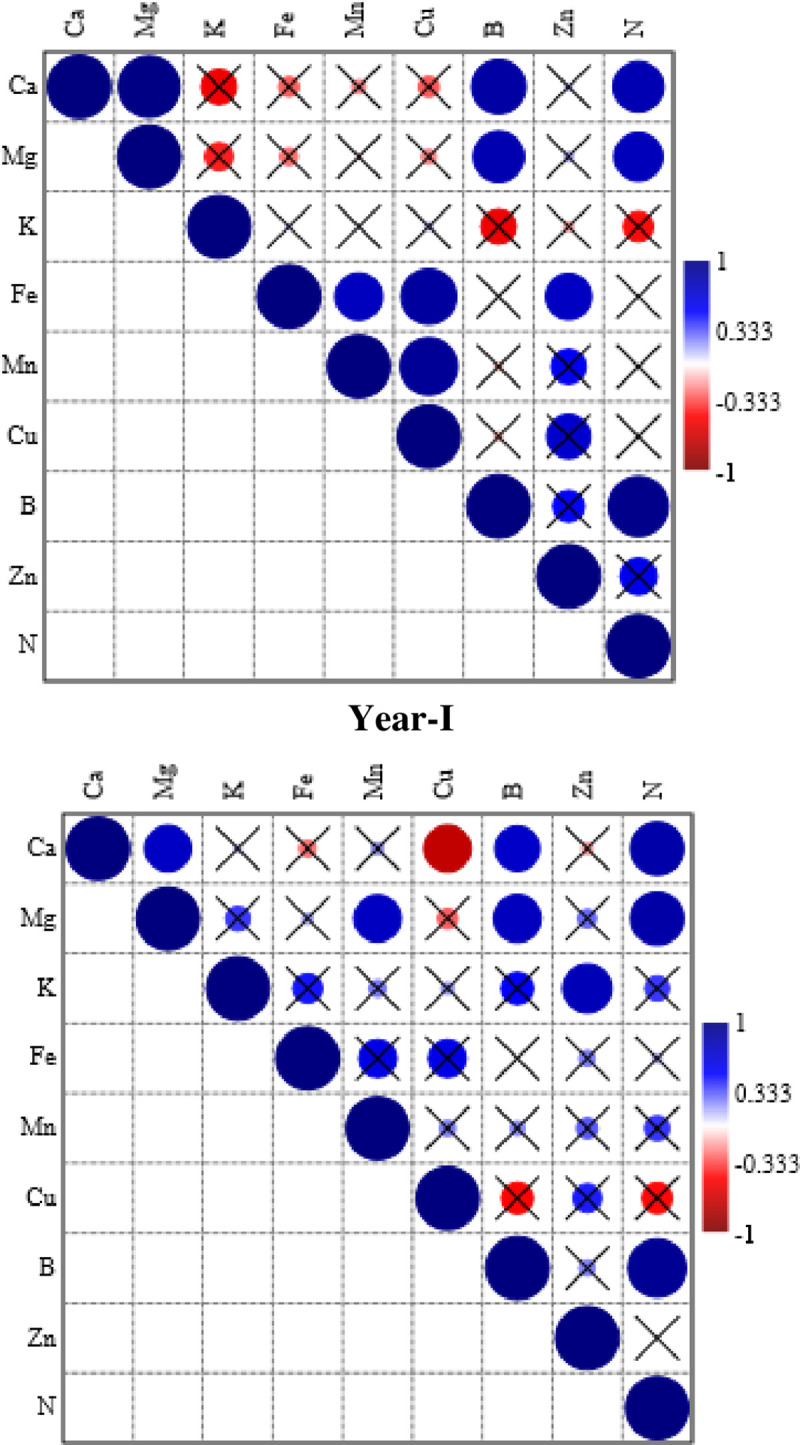
Spearman correlation among different nutrient acquisition traits of graftwed watermelon rind during both years of the study. The size of the circles indicates strength of the correlation, while checked boxes indicate that the correlation was non-significant.

Most of the nutrient uptake traits had non-significant correlations with each other during both years. The only significant and strong positive correlation was noted for Ca and Mg uptake with N accumulation and B during 1^st^ year (Fig 1). Similarly, Fe had significant positive correlation with Zn, and B accumulation was positively correlated with N uptake. Similar correlations were recorded during 2^nd^ year. The only negative correlation was noted among Ca and Cu accumulation during 2^nd^ year (Fig 1).

### Correlation among mineral uptake traits of flesh

**Fig 2:**
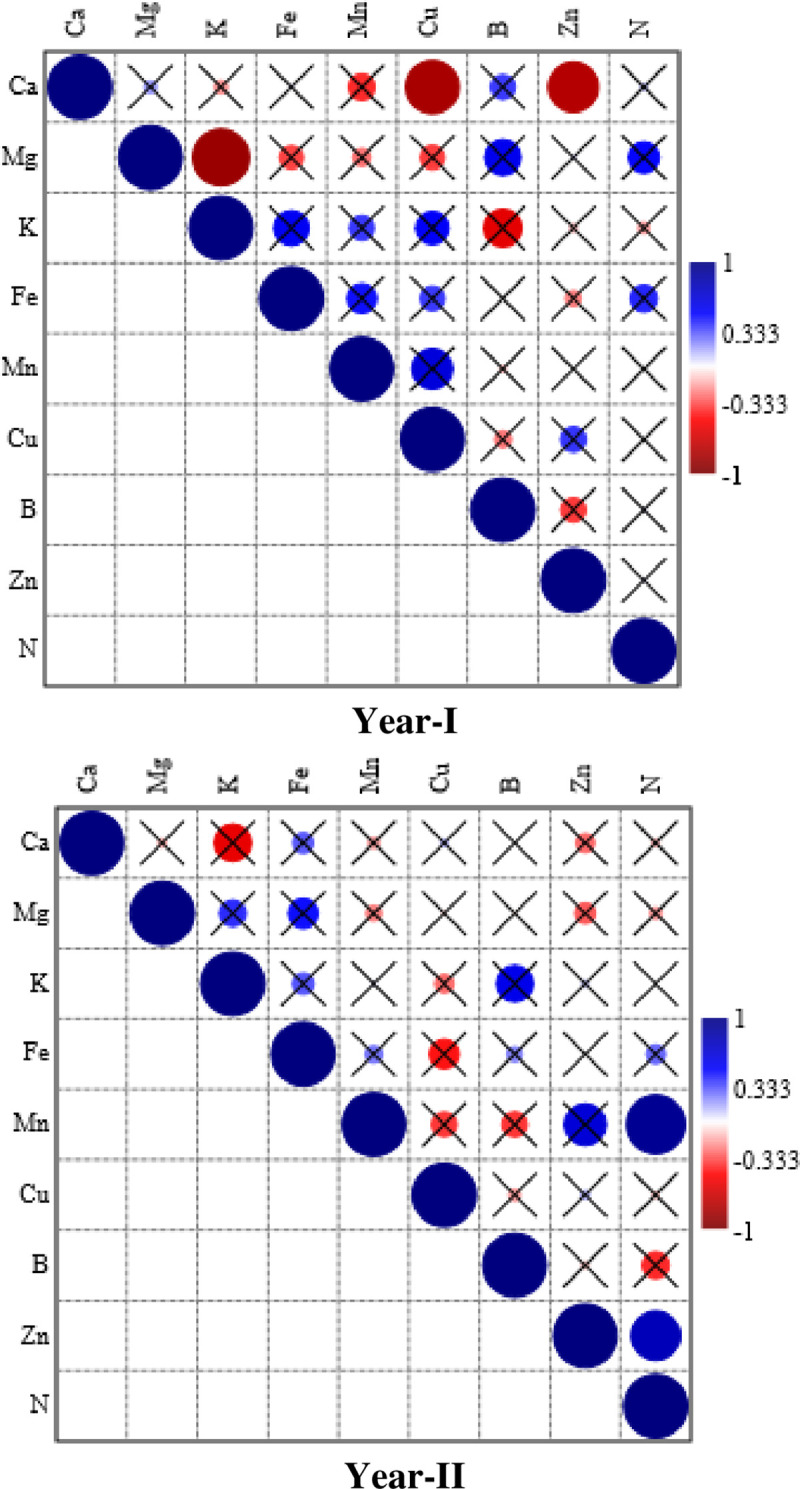
Spearman correlation among different ntrient acquisition traits of graftwed watermelon flesh during both years of the study. The size of the circles indicates strength of the correlation, while checked boxes indicate that the correlation was non-significant.

Most of the nutrient uptake traits had non-significant correlations with each other during both years. The only significant and strong negative correlation was noted for Ca with Cu and Zn uptake during 1^st^ year (Fig 2). The only positive correlation was noted among Mn and N accumulation during 2^nd^ year (Fig 2).

## Discussion

Different nutrient uptake traits were significantly altered by N and B doses. As hypothesized, concentration of most of the nutrients was increased with increasing N and B doses. It was noted that N and other minerals’ concentration increased with N application compared to control treatment. Torun [3] reported that yield, weight, diameter and TSS content of the fruit increased with N application. Similar results have been demonstrated by other researchers [2,46]. Colla et al. [2] also reported that N use efficiency and N uptake efficiency were significantly affected by combinations of N fertilization and grafting. Wehner [47] reported that TSS content in watermelon should be at least 10% for an ideal flavor.

Watermelon is an important fruit vegetable commercially cultivated worldwide. Boron deficiency is common in cultivated areas, globally [17]. Boron fertilizers are used to overcome B-deficiency, which increase input cost. Boron deficiency restricts plant growth and a wide range of symptoms, including chlorosis and thick curled leaves with water soaked black spots appear on watermelon [5]. The adaptability of crops under limited B availability can be attributed to plant ability to absorb B under B-deficient conditions [18].

Nitrogen is required by plants in large amounts for normal growth and development. Numerous metabolic and biochemical process require N for the proper development and yield [6–10]. Low N availability hampers plant growth as it is an important constituent of amino acids, nucleic acid, proteins, chlorophyll and hormones [11].

Boron is widely distributed in earth crust and equally important for plants and animals. The involvement of B in several physiological processes of plants has been reported [21,27,48]. Sufficient B availability in soil solution is important for proper physiological functioning of plants. Principally, B is involved in cell wall structural integration and linkage of B with pectic polysaccharide rhamnogalacturonan II (RGII) controls porosity and tensile strength of cell wall [49]. Considering plant requirement on molar basis, B requirement for dicots is higher compared with any other microelement [48]. However, limitation or excess of B adversely affect plant growth. Interestingly, the range between deficiency and toxicity of B is narrow [50–53]. In soils, the concentration of B varies from 10 mg kg^−1^ to 300 mg kg^−1^ depending on the soil type, amount of organic matter and precipitation [54]. In heavy textured soils B reaches to toxic level that adversely affects plant growth and yield [55,56]. However, in acidic soils B-deficiency is commonly observed because of ion leaching. Boron deficiency alters plant metabolic, cellular, biological and molecular processes such as photosynthesis, cell wall and membrane integration, cell division, carbohydrate metabolism, sugar and hormonal transport, protein biosynthesis and nucleic acid metabolism [27,57]. The obvious response of B-deficiency in several crops is inhibition of root growth because of reduced cell division [58]. Moreover, long-term B-deficiency provokes lipid peroxidation and reduces antioxidant enzymes’ activities because of increased production of reactive oxygen species [48,52].
